# Impact of monocyte to high-density lipoprotein ratio on prevalent hyperuricemia: findings from a rural Chinese population

**DOI:** 10.1186/s12944-020-01226-6

**Published:** 2020-03-16

**Authors:** Meng-Qi Chen, Wen-Rui Shi, Chu-Ning Shi, Ya-Ping Zhou, Ying-Xian Sun

**Affiliations:** 1 Department of Cardiology, The First Hospital of China Medical University, 155 Nanjing North Street, Heping District, Shenyang, 110001 China; 2 Department of Neurology, The First Hospital of China Medical University, 155 Nanjing North Street, Heping District, Shenyang, 110001 China

**Keywords:** Hyperuricemia, Monocyte to high-density lipoprotein ratio, Inflammation, Chinese population

## Abstract

**Background:**

Monocyte to high-density lipoprotein cholesterol ratio (MHR) is a novel inflammatory marker that has been used to predict various inflammation-related diseases. This study aims to explore the association between MHR and prevalent hyperuricemia in a rural Chinese population.

**Methods:**

8163 eligible participants (mean age: 54.13 years, males: 45.71%) from northeast China were enrolled in this cross-sectional study between 2012 to 2013. MHR was determined as blood monocyte count ratio to high-density lipoprotein cholesterol concentration.

**Results:**

The prevalence of hyperuricemia was 12.86%. After adjusting for potential confounding factors, per SD increase of MHR caused a 25.2% additional risk for hyperuricemia, and the top quartile of MHR had an 82.9% increased risk for hyperuricemia compared with the bottom quartile. Additionally, smooth curve fitting and subgroup analyses showed a linear and robust association between MHR and prevalent hyperuricemia respectively. Finally, after introducing MHR into the established model of risk factors, the AUC displayed a significant improvement (0.718 vs 0.724, *p* = 0.008). Furthermore, Category-free net reclassification improvement (0.160, 95% CI: 0.096–0.224, *P* < 0.001) and integrated discrimination improvement (0.003, 95% CI: 0.002–0.005, *P* < 0.001) also demonstrated significant improvements.

**Conclusions:**

The present study suggests that MHR was positively and independently correlated with prevalent hyperuricemia among rural Chinese adults. Our results also implicate an important value for MHR in optimizing the risk stratification of hyperuricemia.

## Background

In recent decades, hyperuricemia is becoming a major public health issue due to its close association with critical diseases such as gout, hypertension, diabetes mellitus, chronic kidney disease and other cardiovascular diseases (CVD) [1–6]. For example, the Mendelian randomization study supported that hyperuricemia may play a causal role in the development of CVD, suggesting its great significance for early screening and prevention of CVD [5]. Accordingly, there is a clear need for a simple approach to improve the risk stratification and prevention of hyperuricemia.

Chronic inflammation is a pathophysiological process characterized by elevated inflammatory mediators which closely associate with hyperuricemia [7, 8]. Epidemiological reports have demonstrated a significant correlation between inflammation and elevated uric acid level [2, 9]. Consistently, previous studies have shown that hyperuricemia might induce inflammation by activating the expression of inflammatory mediators [10, 11]. Therefore, these studies indicated that hyperuricemia was closely related to inflammation.

Monocytes and high-density lipoprotein cholesterol (HDL-C) are two important factors in the development of inflammation [12–14]. Monocytes interact mainly with platelets and endothelial cells, leading to aggravation in inflammation and prothrombotic pathways [15, 16]. In contrast, HDL-C protects endothelial cells from oxidative stress and inflammation by regulating monocytes activation and monocyte progenitors proliferation, and preventing monocytes assembly into the arterial wall [12–14]. High monocyte counts and low HDL-C levels have been shown to be positively associated with inflammation [17–19]. Therefore, while monocytes play a pro-inflammatory role, HDL-C functions as a reverse factor in the inflammatory process.

Monocyte-to-HDL-C ratio (MHR) has been proposed as a potentially modifiable marker of inflammation [20]. Additionally, previous studies have found that MHR has the capacity to predict a variety of inflammation-related diseases [21–24]. As yet, no study has been conducted on the potential association between MHR and the prevalent hyperuricemia. Therefore, our study used data from the Northeast China Rural Cardiovascular Health Study (NCRCHS) to investigate this relationship and explore the value of MHR to optimize the risk stratification of hyperuricemia.

## Methods

### Study population

**Fig. 1:**
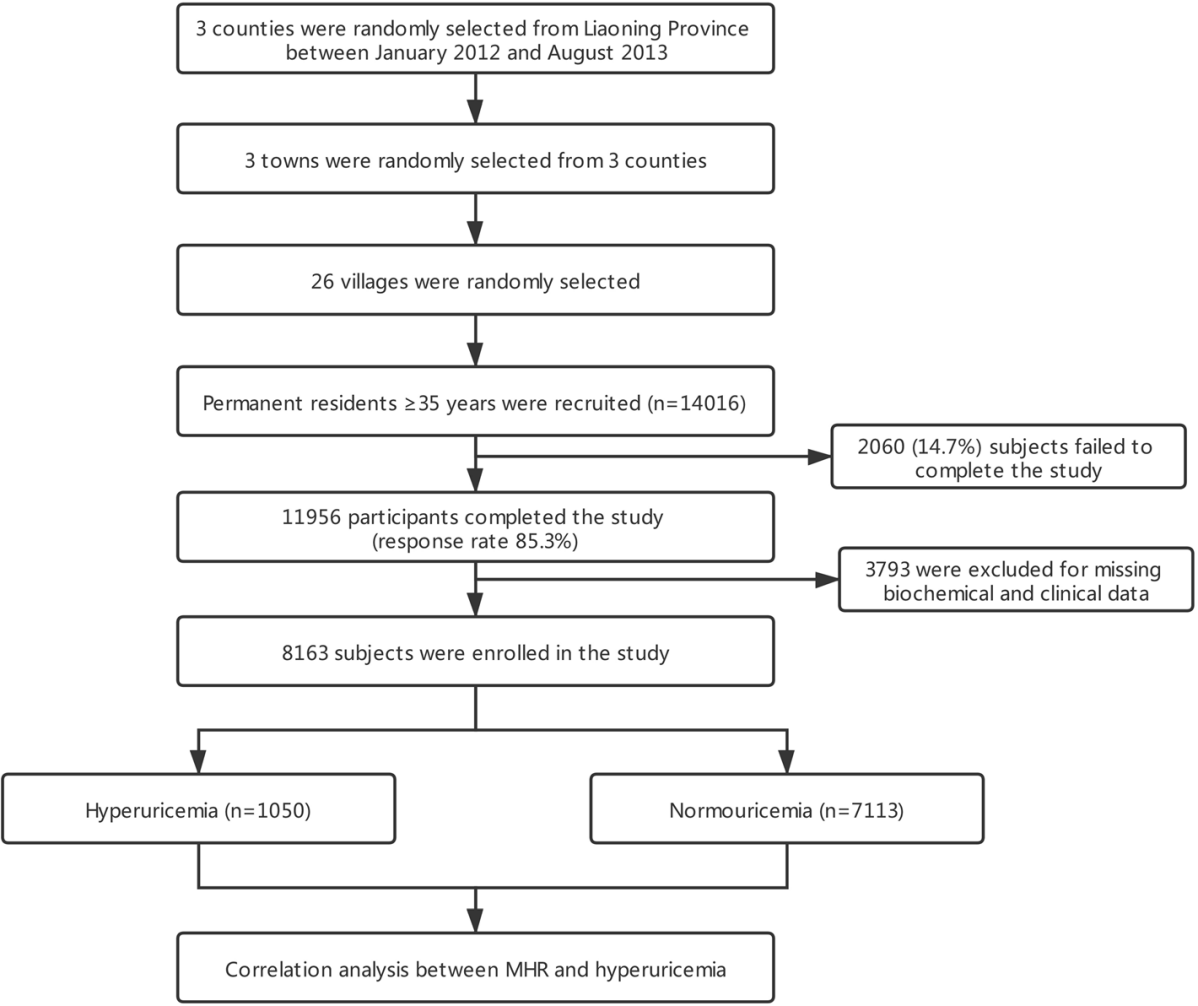
Flowchart describing the recruitment process and generation of study population

The present study was based on a large scale cross-sectional epidemiological survey known as NCRCHS that conducted from January 2012 to August 2013. The detailed design and rationale of NCRCHS were fully described elsewhere [25]. 14,016 permanent residents (age ≥ 35 years) from rural areas of Northeast China were recruited to assess the incidence, prevalence, and natural history of cardiovascular risk factors. Participants were selected by the scheme of a multistage and stratified random sampling. First, three counties of Dawa, Zhangwu, and Liaoyang were selected from the eastern, southern, and northern regions of Liaoning province. Afterward, three towns were randomly selected from three counties. In the end, a total of 26 rural villages were randomly selected. Due to 2060 subjects failed to complete the study, 11,956 individuals were included in our study, producing a response rate of 85.3%. Moreover, 3793 subjects were further excluded for missing biochemical and clinical data. Finally, we enrolled 8163 eligible participants into the present analysis (Fig. 1). Our study was approved by the Ethics Committee of China Medical University (Shenyang, China). Written informed consent was voluntarily signed by all participants; if disabled, informed consent was obtained from the proxies of the subjects.

### Data collection and measurements

Previously published studies have fully reported the methods of data collection and measurement [26, 27]. Before the study, cardiologists and nurses must pass a strict exam to obtain the qualification for conducting questionnaires which collect baseline information about sociodemographic data, health-related behaviors, anthropometric indexes, and history of CVD. Quality assurance of the data collection process was executed by the central steering committee with a subcommittee. The race of participants was separated into Han and others. Education level was split into three categories: primary school or below, middle school, and high school or above. Family annual income of the subjects was classified into three groups: ≤5000, 5000–20,000 and > 20,000 CNY per year. Physical activity was categorized into three levels of low, middle and high based on the self-reports of individuals. Use of lipid-lowering drug was determined as lipid drug uptake over the past 2 weeks. History of CVD included angina pectoris, myocardial infarction, atrial fibrillation, arrhythmia, and heart failure.

The blood pressure of participants was measured three times after at least 5 min of rest in a completely relaxed and sitting position. The result of blood pressure for analysis was the average of three consecutive readings taken by two randomly selected staff.

Concerning anthropometric measurements, individuals were required to wear light clothes without shoes. Calibrated digital scales were applied to measure the standard weight to the nearest 0.1 kg. To quantify standard height with a calibrated stadiometer, subjects were asked to remain standing position. And we recorded the reading of height to the nearest 0.1 cm. Anthropometric measurements were taken twice and the mean values were used into the analysis.

Fasting blood samples of the antecubital veins were gathered in the morning after all participants had fasted for at least 12 h. Venous blood samples were separated into serum samples through a standard centrifuge, and they were transported by EDTA tubes. Finally, the samples were frozen at − 20 C degree for a better analysis of serum creatinine (Scr), fasting plasma glucose (FPG), triglyceride (TG), total cholesterol (TC), low-density lipoprotein cholesterol (LDL-C), HDL-C, white blood cells (WBC), lymphocytes, neutrophils and monocytes.

### Definition

Body mass index (BMI) was calculated as mean weight divided by mean height squared (kg/m2). The estimated glomerular filtration rate (eGFR) was defined according to the CKD-EPI (Chronic Kidney Disease Epidemiology Collaboration) equation [28]. MHR was determined as blood monocyte count ratio to high-density lipoprotein cholesterol concentration [29]. The definition of hyperuricemia was serum uric acid (SUA) ≥ 357 μmol/L (6 mg/dL) for females and ≥ 417 μmol/L (7 mg/dL) for males [30].

### Statistical analysis

Continuous variables were presented as mean values ± standard deviation (SD) or median (interquartile range) based on the distribution. Category variables were displayed as frequencies (percentages). Students’ t-test or Mann-Whitney test was applied to compare continuous variables between groups. Chi-square test was adopted to compare categorical variables between groups. In addition, the rank-sum test was employed to make the utmost of ordinal information for ordinal category variables. Multivariate logistic regression was utilized to demonstrate the independent relationship between MHR and the prevalence of hyperuricemia. Odds ratio (OR) and 95% confidence interval (95% CI) were presented in the results. The linear relationship between normalized MHR and the prevalence of hyperuricemia was explored by a spline smoothing function with a generalized additive model. Subgroup analyses were tested to detect the robustness of the association between MHR and the prevalent hyperuricemia. Finally, receiver operating characteristic (ROC) curve, integrated discrimination improvement (IDI) and category-free net reclassification improvement (NRI) was employed to estimate the potential of MHR to enhance the risk classification of hyperuricemia. The whole statistical analyses were performed by SPSS 25.0 software (IBP corp), EmpowerStats (http://www.empowerstats.com, X&Y Solutions, Inc., Boston, MA) and statistical software packages R (http://www.R-project.org, The R Foundation). Statistical significance was identified by a two-tailed *P* value < 0.05.

## Results

Table 1 summarizes the baseline characteristics of 8163 subjects (45.71% men and 54.29% women). The crude prevalence of hyperuricemia was 12.86%. As for demographic data, population with hyperuricemia were older and had a higher proportion of male as well as the Han race than the healthy group. Furthermore, hyperuricemia participants had lower family annual income and more likely to be a current drinker or smoker compared with normouricemia subjects. About the anthropometric characteristics, hyperuricemia group had significantly higher levels of height, weight, BMI, SBP, and DBP. Laboratory examinations exhibited higher Scr, FPG, TC, TG, and LDL-C concentrations together with greater numbers of white blood cells, lymphocytes, neutrophils and monocytes in the patients’ group. Additionally, hyperuricemia patients had markedly lower eGFR and HDL-C levels. Moreover, the percentages of CVD history, and lipids-lowering drug were statistically augmented in the hyperuricemia subjects. Lastly, we could observe a substantially greater level of MHR in the hyperuricemia group than the normouricemia group (all *P* < 0.05).

**Table 1:** Characteristics of subjects stratified by hyperuricemia

Variables	Total (*n* = 8163)	Hyperuricemia (*n* = 1050)	Normouricemia (*n* = 7113)	*P* value ^a^
Age (years)	54.13 ± 10.49	55.12 ± 10.96	53.99 ± 10.41	0.001
Males (%)	3731 (45.71)	652 (62.10)	3079 (43.29)	< 0.001
Race, Han (%)	7936 (97.22)	1033 (98.38)	6903 (97.05)	0.014
Education (%)				0.716
Primary school or below	4322 (52.95)	561 (53.43)	3761 (52.88)	
Middle school	3134 (38.39)	393 (37.43)	2741 (38.54)	
High school or above	707 (8.66)	96 (9.14)	611 (8.59)	
Income, CNY (%)				0.012
≤ 5000	851 (10.43)	134 (12.76)	717 (10.08)	
5000–20,000	4294 (52.60)	557 (53.05)	3737 (52.54)	
> 20,000	3018 (36.97)	359 (34.19)	2659 (37.38)	
Physical activity (%)				0.101
Low	3192 (39.10)	436 (41.52)	2756 (38.75)	
Middle	1547 (18.95)	205 (19.52)	1342 (18.87)	
High	3424 (41.95)	409 (38.95)	3015 (42.39)	
Current smoking (%)	2767 (33.90)	398 (37.90)	2369 (33.31)	0.003
Current drinking (%)	1734 (21.24)	332 (31.62)	1402 (19.71)	< 0.001
Height (cm)	160.66 ± 8.15	162.97 ± 8.45	160.32 ± 8.05	< 0.001
Weight (kg)	63.49 ± 11.27	69.85 ± 12.16	62.56 ± 10.82	< 0.001
BMI (kg/m^2^)	24.54 ± 3.61	26.26 ± 3.85	24.29 ± 3.50	< 0.001
SBP (mmHg)	138.68 ± 21.93	144.79 ± 23.44	137.77 ± 21.55	< 0.001
DBP (mmHg)	81.86 ± 11.64	86.09 ± 12.63	81.24 ± 11.35	< 0.001
Scr (μmol/L)	73.80 (66.70–82.20)	82.70 (74.73–91.80)	72.80 (66.10–80.70)	< 0.001
eGFR (ml/min per 1.73 m^2^)	89.33 ± 15.42	81.92 ± 19.13	90.42 ± 14.48	< 0.001
FPG (mmol/L)	5.60 (5.22–6.10)	5.72 (5.32–6.35)	5.58 (5.22–6.06)	< 0.001
TC (mmol/L)	5.32 ± 1.11	5.63 ± 1.28	5.27 ± 1.07	< 0.001
TG (mmol/L)	1.29 (0.91–1.96)	1.88 (1.28–2.87)	1.23 (0.88–1.83)	< 0.001
HDL-C (mmol/L)	1.34 ± 0.32	1.28 ± 0.33	1.35 ± 0.32	< 0.001
LDL-C (mmol/L)	2.89 ± 0.80	3.07 ± 0.88	2.87 ± 0.79	< 0.001
WBC count (10^9^/L)	6.20 ± 2.06	6.61 ± 1.79	6.14 ± 2.09	< 0.001
Lymphocyte count (10^9^/L)	1.90 (1.60–2.40)	2.10 (1.70–2.50)	1.90 (1.60–2.30)	0.017
Neutrophil count (10^9^/L)	3.40 (2.70–4.30)	3.70 (2.90–4.60)	3.40 (2.60–4.20)	< 0.001
Monocyte count (10^9^/L)	0.41 (0.30–0.60)	0.50 (0.40–0.70)	0.40 (0.30–0.60)	< 0.001
Lipid-lowering drug (%)	272 (3.33)	78 (7.43)	194 (2.73)	< 0.001
History of CVD (%)	1268 (15.53)	222 (21.14)	1046 (14.71)	< 0.001
SUA (μmol/L)	300.77 ± 85.68	452.12 ± 70.17	278.43 ± 61.79	< 0.001
MHR	0.38 ± 0.25	0.45 ± 0.27	0.37 ± 0.24	< 0.001

Data are expressed as mean ± standard deviation (SD) or median (interquartile range) and numbers (percentage) as appropriate

*Abbreviations*: *CNY* Chinese currency (1CNY = 0.15 USD), *BMI* body mass index, *SBP* systolic blood pressure, *DBP* diastolic blood pressure, *Scr* serum creatinine, *eGFR* estimated glomerular filtration rate, *FPG* fasting plasma glucose, *TC* total cholesterol, *TG* triglyceride, *HDL-C* high-density lipoprotein cholesterol, *LDL-C* low-density lipoprotein cholesterol, *WBC* white blood cell, *CVD* cardiovascular disease, *SUA* serum uric acid, *MHR* Monocyte to high-density lipoprotein ratio

^a^Comparisons for category variables between groups were tested by χ2 test or rank-sum test (ordinal category variables) and comparisons of continuous variables between groups were tested by Student’s t-test or Mann-Whitney test

Logistic regression analyses revealed the association between MHR and prevalent hyperuricemia, as displayed in Table 2. In model 2, per SD increase of MHR caused a 37.5% additional risk for hyperuricemia after adjusting for age, sex, race, education level, family annual income and physical activity, current smoking and drinking status. After additional adjustment of BMI, eGFR, TC, HDL-C, SBP, FPG, lipid-lowering drug, and CVD history, the risk attenuated to 25.2%. When dividing MHR into quartiles, we could observe the top quartile had an 82.9% increased risk for hyperuricemia compared with the bottom quartile in the fully adjusted model. Furthermore, the prevalence of hyperuricemia displayed a significant linear trend across the quartiles (P for trend < 0.001).

**Table 2:** Multivariate logistic regression of MHR for hyperuricemia

Variables	Odds Ratio (95%CI)					
	Model 1	*P* value	Model 2	*P* value	Model 3	*P* value
MHR (per SD change)	1.394 (1.299, 1.496)	< 0.001	1.375 (1.280, 1.478)	< 0.001	1.252 (1.157, 1.355)	< 0.001
Quartiles of MHR						
Quartile 1	Reference		Reference		Reference	
Quartile 2	1.272 (1.035, 1.564)	0.022	1.243 (1.009, 1.531)	0.041	1.194 (0.963, 1.480)	0.106
Quartile 3	1.447 (1.183, 1.771)	< 0.001	1.392 (1.135, 1.708)	0.002	1.264 (1.019, 1.569)	0.033
Quartile 4	2.418 (2.001, 2.920)	< 0.001	2.320 (1.913, 2.813)	< 0.001	1.829 (1.477, 2.264)	< 0.001
P for trend		< 0.001		< 0.001		< 0.001

Model 1: no adjustment; Model 2: adjusted for age, sex, race, education level, family annual income, physical activity, current smoking, current drinking; Model 3: adjusted for all the factors in model 2 and BMI, eGFR, TC, HDL-C, SBP, FPG, lipid-lowering drug, history of CVD

*Abbreviations*: *MHR* Monocyte to high-density lipoprotein ratio, *OR* odds ratio, *CI* confidence interval, *SD* standard deviation. Other abbreviations as in Table 1

Quartile 1: MHR < 0.227; Quartile 2: 0.227 ≤ MHR < 0.339; Quartile 3: 0.339 ≤ MHR < 0.484; Quartile 4: MHR ≥0.484

**Fig. 2:**
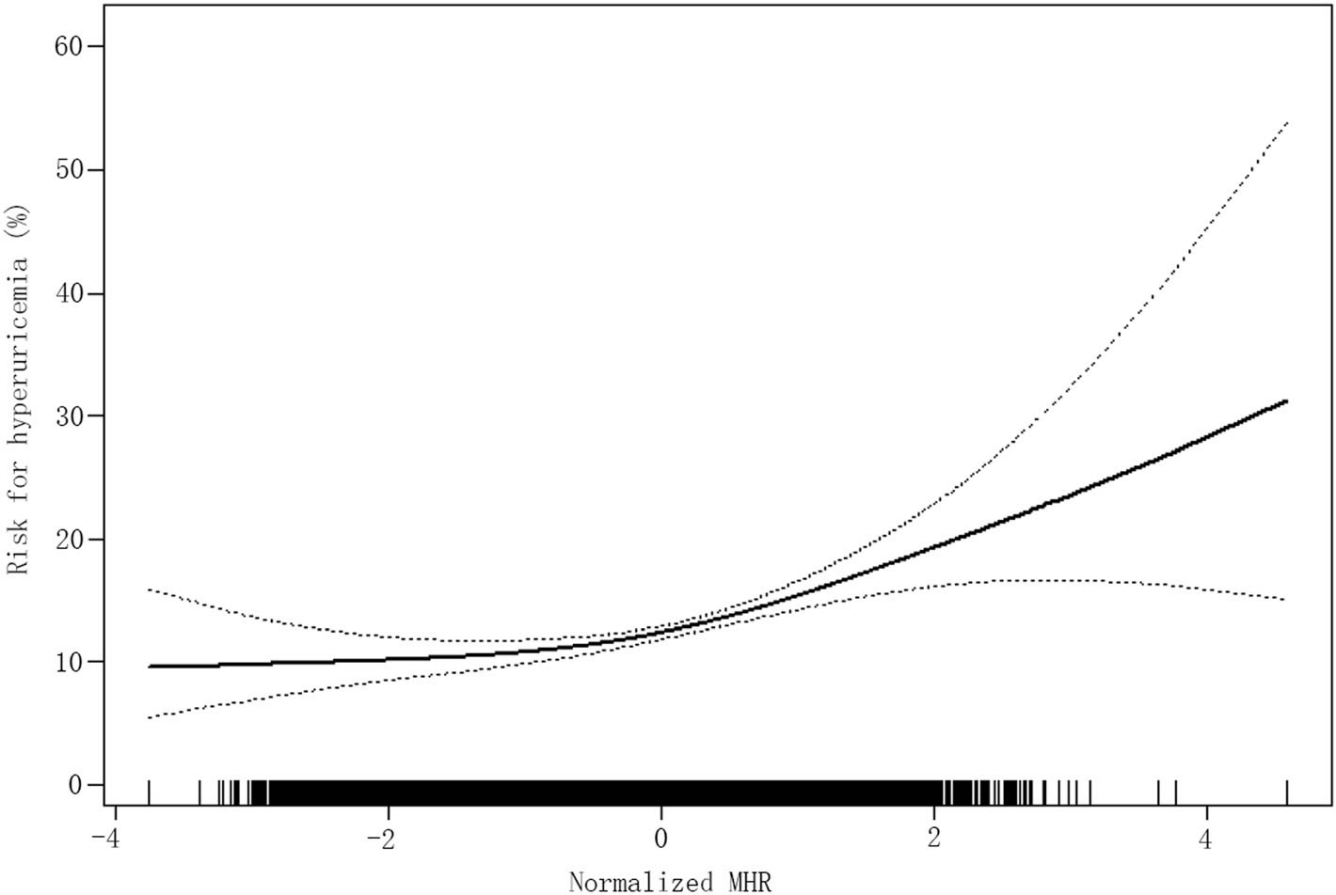
Smooth curve fitting was employed using generalized additive model to investigate the association between MHR and the risk of hyperuricemia after adjusting for age, sex, race, education level, family annual income, physical activity, current smoking, current drinking, BMI, eGFR, TC, HDL-C, SBP, FPG, lipid-lowering drug, history of CVD. In the figure, the solid line represents the estimated risk of hyperuricemia, and the dotted line indicates pointwise 95% confidence interval

To further demonstrate the linear association between MHR and the prevalence of hyperuricemia, we performed a smooth curve fitting with full adjustment of all covariates (Fig. 2). The curve showed a linear correlation between normalized MHR and the risk of hyperuricemia, and the result confirmed the linear trend in the above-described quartile analysis of logistic regression.

**Fig. 3:**
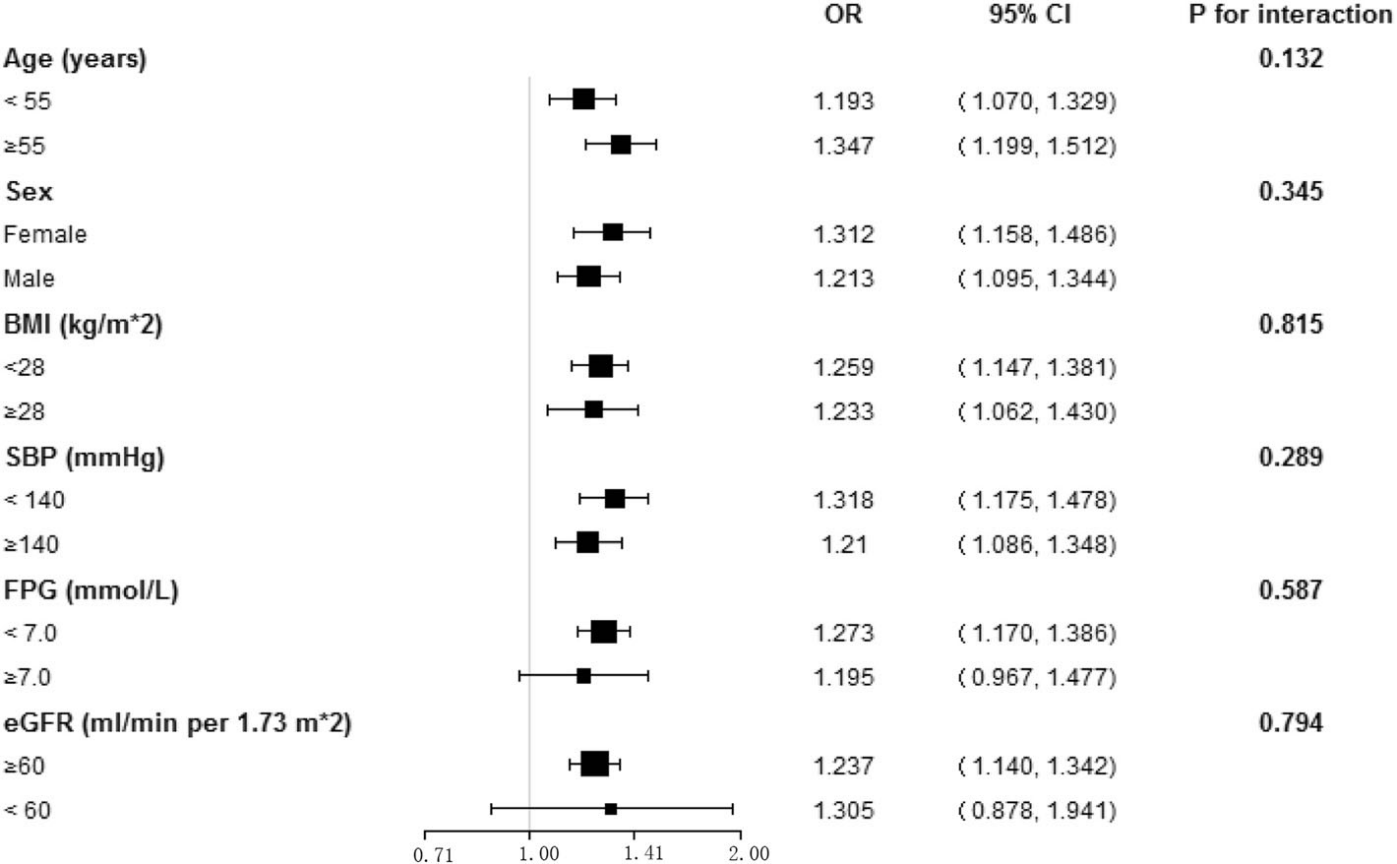
Subgroup analysis for the impact of MHR on the prevalence of hyperuricemia. The dots and lines represent the estimates of the odds ratios of hyperuricemia for per SD increment of MHR and the corresponding 95% confidence intervals, respectively. The model adjusted for sex, age, race, education level, family annual income, physical activity, current smoking, current drinking, BMI, eGFR, TC, HDL-C, SBP, FPG, lipid-lowering drug, history of CVD, except for the variable that is stratified

To investigate whether the relationship between MHR and hyperuricemia was robust in the logistic regression model, stratified analyses were conducted using several identified risk factors (including age, sex, BMI, SBP, FPG, and eGFR) for hyperuricemia (Fig. 3). After adjusting for the above-described covariates except for the covariate used for stratification, the results of the subgroup analyses revealed the robust association between MHR and hyperuricemia (all P for interaction > 0.05).

Finally, through ROC analysis and reclassification analyses, the results illuminated the ability of MHR to optimize the risk stratification of hyperuricemia (Table 3). The AUC of MHR for hyperuricemia was 0.598 (95% CI: 0.587–0.608, *P* < 0.001), and the AUC of the model of several clinical risk factors (including age, sex, current smoking, current drinking, BMI, eGFR, TC, HDL-C, SBP, FPG and history of CVD) was 0.718 (95% CI: 0.709–0.728, *P* < 0.001). When adding MHR into clinical risk factors, we could detect a statistical increment of AUC (0.724, 95% CI: 0.715–0.734, *P* < 0.001). Additionally, both the category free NRI (0.160, 95% CI: 0.096–0.224, *P* < 0.001) and IDI (0.003, 95% CI: 0.002–0.005, *P* < 0.001) showed a significant improvement when adding MHR into above risk factors.

**Table 3:** Receiver operating characteristic and reclassification analyses for MHR to discriminate the risk stratification of hyperuricemia

Model	AUC (95% CI)	*P* value	P for comparison	NRI (category free)	*P* value	IDI	*P* value
MHR	0.598 (0.587, 0.608)	< 0.001	–	–	–	–	–
Clinical risk factors*	0.718 (0.709, 0.728)	< 0.001	Reference	Reference	Reference	Reference	Reference
Clinical risk factors + MHR	0.724 (0.715, 0.734)	< 0.001	0.008	0.160 (0.096, 0.224)	< 0.001	0.003 (0.002, 0.005)	< 0.001

* Clinical risk factors: age, sex, current smoking, current drinking, BMI, eGFR, TC, HDL-C, SBP, FPG, history of CVD

*Abbreviations*: *MHR* Monocyte to high-density lipoprotein ratio, *AUC* area under the curve, *CI* confidence interval, *NRI* net reclassification improvement, *IDI* integrated discrimination improvement, *BMI* body mass index, *eGFR* estimated glomerular filtration rate, *TC* total cholesterol, *HDL-C* high density lipoprotein cholesterol, *SBP* systolic blood pressure, *FPG* fasting plasma glucose, *CVD* cardiovascular disease

## Discussion

Our study for the first time implicated the impact of MHR on the prevalent hyperuricemia in the general population. Furthermore, the findings revealed the association between MHR and the prevalence of hyperuricemia was linear and robust. Additionally, our analyses suggest that MHR may have the capacity to optimize the risk stratification of hyperuricemia. Taken together, our findings suggest the strong association between MHR and the prevalent hyperuricemia, and the value of MHR to optimize the risk stratification of hyperuricemia.

Monocyte counts and HDL-C levels are two pivotal hallmarks in the development and progression of inflammation [12–14]. Chronic inflammation is a systematic process accompanied by an elevation of inflammatory mediators such as acute-phase proteins, cytokines, and adhesion molecules [7]. Monocytes are crucial immune system cells that play a unique role in the inflammatory response [13, 31]. Activated monocyte interacts with activated or damaged endothelial cells, leading to overexpression of proinflammatory cytokines and adhesion molecules, including vascular cell adhesion molecule 1, monocyte chemotactic protein 1 ligand and intercellular adhesion molecule 1. Thereafter, monocytes move to the subendothelial space and then differentiate into macrophages that engulf oxidized low-density lipoprotein cholesterol [32]. These cells then transform into foam cells that release pro-inflammatory cytokines, attracting more monocytes into the site of inflammation [13, 33]. However, HDL-C plays a key role in the anti-inflammatory effect. HDL-C counteracts the proinflammatory response of monocytes by interrupting the differentiation of monocyte to macrophage and preventing the recruitment of monocyte into vascular wall, as well as prohibiting LDL-C oxidation in the arterial wall [14, 34]. Additionally, HDL-C inhibits the proliferation of monocyte progenitor cells [35]. Therefore, monocytes exhibit proinflammatory effects, but HDL-C acts as a reverse factor in the process of inflammation.

MHR as the combination of both monocytes and HDL-C has been proposed as a novel inflammatory index [20]. Recent clinical and epidemiological studies have established that MHR has the ability to predict multiple inflammation-related diseases, such as metabolic syndrome, diabetes mellitus, atrial fibrillation and coronary artery disease [21–24]. Recent researches have revealed the significant association between inflammation and elevated uric acid level [2, 9]. For example, C-reactive protein (CRP), a marker of low-grade inflammation, was positively correlated with SUA levels [36, 37]. Furthermore, uric acid can induce CRP mRNA expression in vascular endothelium and smooth muscle cells [38]. Hence, these studies indicated a close relationship between SUA and inflammation. In view of the foregoing, we hypothesize that MHR, a novel inflammatory index, also has a significant association with hyperuricemia and the ability to improve risk stratification of hyperuricemia.

The current findings were consistent with our hypothesis. Regardless of whether MHR was used as a continuous variable or a category variable, the results of the logistic regression analysis showed a significant positive correlation between MHR and hyperuricemia. Moreover, we further testified the linear association of MHR with hyperuricemia via performing smooth curve fitting analysis. Therefore, higher MHR suggests a proportionally higher prevalence of hyperuricemia, excluding the presence of threshold or saturation effects. Additionally, in the subgroup analyses of age, sex, BMI, SBP, FPG and eGFR, the stable relationship between MHR and hyperuricemia suggests that the risk stratification capacity of MHR is applicable to these designated populations.

Recently, a substantial proportion of studies reported that serum uric acid was independently associated with chronic kidney diseases [39–42], and it has been demonstrated that MHR was independently associated with reduced renal function [43]. However, our study found that there was not a statistically significant correlation between the MHR and the prevalent hyperuricemia in individuals with an eGFR of less than 60 ml/min per 1.73 m^2^. The main reason may be the decrease of statistic power because of the small sample size of participants with hyperuricemia and reduced renal function simultaneously. Similar to our finding, one Mendelian randomization study, using uric acid transporter genetic risk score to explore the causality between serum uric acid and kidney function, suggested that a possible causal relationship between serum uric acid levels and improved renal function in healthy men rather than and reduced renal function [44]. In any case, further study is needed to confirm the current results.

ROC and reclassification analyses were applied to evaluate the risk stratification ability of MHR for hyperuricemia. In the ROC analysis, MHR as a single indicator had a statistically significant identification of hyperuricemia, but the AUC value was too low to be practically applied, and then we incorporated MHR into a clinical risk factors model and the results showed a significant improvement in risk identification of hyperuricemia (0.718 vs 0.724, *p* = 0.008). To further confirm the capacity of MHR to optimize the risk stratification of hyperuricemia, we conducted reclassification analysis including both IDI and NRI which can assess the incremental potential of adding a new risk marker into an established risk model [45, 46]. As expected, both results of category-free NRI and IDI presented a significant improvement in stratifying hyperuricemia risk when MHR was introduced into the established model of risk factors. In summary, our results suggest that MHR has the ability to optimize the risk stratification of hyperuricemia.

This study exists several limitations, which should be taken into account when considering the results. The first disadvantage is that cross-sectional design can only suggest the correlation between MHR and hyperuricemia, but the causality of this association needs to be confirmed by further prospective studies. Second, the study participants were enrolled from 26 rural areas in northeastern China, therefore, whether our results are applicable to the general Chinese population still deserves more studies to evaluate. Third, possible unmeasured confounding variables may exist and could have affected the results. The present study has adjusted age, sex, race, education level, family annual income, physical activity, current smoking, current drinking, BMI, eGFR, TC, HDL-C, SBP, FPG, lipid-lowering drug, and history of CVD, all of which are potential confounding, because they affect both exposure and outcome. However, there may be some variables that were not included in our analyses but can affect hyperuricemia, such as gout and hypouricemic drugs. Finally, considering the economic feasibility of epidemiological study, traditional inflammation markers such as CRP, ferritin and interleukin 6, which could be used to compare the predictive power with MHR for hyperuricemia, were not collected in present study. Therefore, whether MHR is a better predictor for hyperuricemia than traditional inflammation markers still needs further studies and evaluation.

## Conclusions

In conclusion, MHR as a novel and simple marker of inflammation was independently associated with the prevalent hyperuricemia in a rural Chinese population. Our results also suggest the important value of MHR to optimize the risk stratification and prevention of hyperuricemia. However, our results should be verified in large prospective studies to explain the definite mechanism of MHR in hyperuricemia.

## Data Availability

The datasets used and/or analyzed during the current study are available from the corresponding author on reasonable request.

## Abbreviations

MHR: Monocyte to high-density lipoprotein ratio
SUA: Serum uric acid
CNY: Chinese currency
CRP: C-reactive protein
CKD-EPI: Chronic Kidney Disease Epidemiology Collaboration
BMI: Body mass index
SBP: Systolic blood pressure
DBP: Diastolic blood pressure
Scr: Serum creatinine
eGFR: Estimated glomerular filtration rate
FPG: Fasting plasma glucose
TC: Total cholesterol
TG: Triglyceride
HDL-C: High-density lipoprotein cholesterol
LDL-C: Low-density lipoprotein cholesterol
WBC: White blood cell
CVD: Cardiovascular disease
NCRCHS: Northeast China Rural Cardiovascular Health Study
OR: Odds ratio
EDTA: Ethylenediaminetetraacetic acid
CI: Confidence interval
SD: Standard deviation
AUC: Area under the curve
CI: Confidence interval
NRI: Net reclassification improvement
IDI: Integrated discrimination improvement
